# The London Hospitals

**Published:** 1902-06-21

**Authors:** 


### The Institutional Workshop.

### THE LONDON HOSPITALS.

### MESSAGES FROM GREAT PERSONAGES.

It is distinctly encouraging to the hospitals, and their maintenance upon a voluntary basis, that the following eminent persons should have promptly set aside other matters and immediately consented to write a short appeal on their behalf in connection with Hospital Sunday in London in the Coronation Year. It will be observed, as one of our contemporaries is shrewd enough to point out, that the short list of eminent personages to whom our request was addressed includes leaders in the Church, politics, law, the Army, literature, and the drama. No message was received from any member of the Liberal party—possibly because we failed to discover the leaders of that party—or from the medical profession, although we took infinite pains to extract a few lines in support of the hospitals from Sir Frederick Treves, who was apparently too busy to even acknowledge the receipt of the communications addressed to him in the cause of charity. It was very difficult to determine which member of the medical profession would appeal most directly to the outside public, and we selected Sir Frederick Treves as owing to the lamented death of Sir W. MacCormac he probably represented most closely in the public mind the topics of the moment which, next to the Coronation, were undoubtedly the war and the peace. Our complete failure to extract even a few lines on behalf of all the hospitals from this distinguished surgeon illustrates one of the causes which make some people hesitate to support hospitals in the present day. Our hospitals can only secure the funds which they are entitled to claim from the public by the heartfelt, earnest co-operation of the leaders of public opinion in all ranks of life. It is not a little remarkable that the great medical profession should in the circumstances stated have remained dumb at a time like the present when a supreme effort has been made to place the London hospitals once and for all on a sound financial basis.

Our failure to elicit even a reply from Sir Frederick Treves makes the generosity and public spirit of the course taken by the Lord Chief Justice, the Leader of the House of Commons, the Secretary of State for the Colonies, the Archbishop of Canterbury, the Commander-in-Chief, the most representative resident member of our Empire beyond the seas—Lord Strathcona—great writers like the three whose names appear below, and leaders of the drama in the persons of Sir Henry Irving and Mr. H. Beerbohm Tree, the more noteworthy and grateful. We have reason to know—indeed everybody must know—the enormous pressure of work which these great men are subjected to at the present moment especially, and their generous co-operation will never be forgotten by the hospitals nor by their countrymen, whose appreciation was well voiced by one of the preachers on Hospital Sunday, who declared "that everybody must feel how very much all who are interested in the hospitals owe to them for rousing the public to a sense of their duty, and, what is even more important, guiding them in exhibiting that interest which without guidance is so fatal."

Lord Salisbury, Lord Kelvin, Lord Lister, Lord Rosebery, the Baroness Burdett-Coutts, the Poet Laureate, Mrs. Kendal, Mr. Rudyard Kipling, and Lucas Malet were unable to write anything this year.

The following is a complete record of the messages received on behalf of the hospitals :—

The Archbishop of Canterbury : "With all my heart I commend the work of supporting the hospitals to the people of London. I trust that everything possible may be done to help them in their work—the noblest work that man can do."

The Right Hon. A. J. Balfour : "I have already spoken on behalf of the London hospitals, and can do little more than repeat what I have already said. They provide the most powerful machinery at our disposal for the alleviation of suffering, and it is to them we must largely look to supply the medical training and the opportunities for research on which the future of medicine so greatly depends. They are of as much importance, therefore, to the rich as to the poor, to the future as to the present, and should be regarded as in the fullest sense objects of national concern."

The Right Hon. Joseph Chamberlain : "I heartily wish you success in your effort of mercy, which, at this time of general thanksgiving and rejoicing, will specially appeal to the British public."

Field-Marshal Earl Roberts : "I trust that Coronation Hospital Sunday will be made memorable by a ready and generous response to the appeal upon behalf of the London hospitals, and that the large sum required to meet their urgent needs will be forthcoming. I know so well how much our London hospitals have done in regard to providing medical and nursing service for our soldiers during the recent war, and at this time of national joy and thanksgiving for the blessings of peace we should do all in our power to alleviate the sufferings of the poor."

The Lord Chief Justice : "It is in my opinion quite impossible to exaggerate the value of the work done by the London hospitals. They minister from day to day to those classes of the population which, without their aid, would be left to bear untended the greatest suffering, and anyone who has experience of their work knows that the highest skill and the greatest attention is at the disposal of the poorest of the land. I will gladly do anything in my power to press their claims upon the charitable and beneficent."

Lord Strathcona : "No words could more eloquently appeal to the public than your statement that the London hospitals annually administer to the wants of some two millions of the sick and ailing, who are also poor and needy."

Mr. I. Zangwill : "Who would be most truly loyal to the King will respond to that which is most truly royal in the King—his Majesty's care for the poorest of his subjects. The daily struggle for life yields grimmer casualty lists than any battlefield, and it is a war in which peace can never be proclaimed. Let us at least ensure that the Red Cross corps of Peace shall be efficient and unfailing."

Mrs. Craigie ("John Oliver Hobbes"): "At this season of thanksgiving and festivals, it is an especial happiness and a peculiar duty to remember that we all have an opportunity on Coronation Hospital Sunday to help the disabled multitude of those who will rejoice under pain, anxiety, and illness at the London hospitals, which are in reality the hospitals of the whole Empire."

Dr. Conan Doyle: "Best wishes; great success; should think those who wish to give thank-offering for peace could not possibly do better than in helping this noble work."

Sir Henry Irving: "The hospital work is noble work, and should have the help of all loyal souls at this time of the nation's double rejoicing."

Mr. H. Beerbohm Tree: "I feel sure I am only echoing the sentiments of all the members of my calling in hoping that your appeal next Sunday may meet with a generous and general response. To help the hospitals the actors have always done their utmost, and they always will. It is not so wonderful that those whose work it is to afford pleasure should be in hearty sympathy with those whose mission it is to alleviate pain. May your great work of merciful charity find all help and encouragement."

